# Long-Distance Protonation-Conformation Coupling in Phytochrome Species

**DOI:** 10.3390/molecules27238395

**Published:** 2022-12-01

**Authors:** Maryam Sadeghi, Jens Balke, Timm Rafaluk-Mohr, Ulrike Alexiev

**Affiliations:** Department of Physics, Freie Universität Berlin, Arnimallee 14, 14195 Berlin, Germany

**Keywords:** phytochrome, Agp1, Cph1, biliverdin, chromophore protonation, conformational coupling

## Abstract

Phytochromes are biological red/far-red light sensors found in many organisms. The connection between photoconversion and the cellular output signal involves light-mediated global structural changes in the interaction between the photosensory module (PAS-GAF-PHY, PGP) and the C-terminal transmitter (output) module. We recently showed a direct correlation of chromophore deprotonation with pH-dependent conformational changes in the various domains of the prototypical phytochrome Cph1 PGP. These results suggested that the transient phycocyanobilin (PCB) chromophore deprotonation is closely associated with a higher protein mobility both in proximal and distal protein sites, implying a causal relationship that might be important for the global large-scale protein rearrangements. Here, we investigate the prototypical biliverdin (BV)-binding phytochrome Agp1. The structural changes at various positions in Agp1 PGP were investigated as a function of pH using picosecond time-resolved fluorescence anisotropy and site-directed fluorescence labeling of cysteine variants of Agp1 PGP. We show that the direct correlation of chromophore deprotonation with pH-dependent conformational changes does not occur in Agp1. Together with the absence of long-range effects between the PHY domain and chromophore p*K*_a_, in contrast to the findings in Cph1, our results imply phytochrome species-specific correlations between transient chromophore deprotonation and intramolecular signal transduction.

## 1. Introduction

Phytochromes are photoreceptor proteins and belong to the class of bilin-containing proteins. Phytochromes control many developmental processes in plants such as seed germination, de-etiolation, or flowering [1]. The discovery of the bacterial phytochrome Cph1 [2] indicates the prokaryotic origin of these photoreceptor proteins, which include prototypical and bathy phytochromes. Prototypical phytochromes, including Cph1 from the cyanobacterium *Synechocystis* 6803 [2] and Agp1 from the soil bacterium *Agrobacterium tumefaciens* [3], act as photochemical switches that interconvert between stable red (Pr)- and metastable far-red (Pfr)-absorbing states. This interconversion is induced by photoisomerization of the bilin chromophore after light activation [4,5]. The bilin chromophore deprotonates transiently during the Pr to Pfr photoconversion in association with extensive global structural changes required for signal transmission [6]. Bathy phytochromes, including Agp2 from *Agrobacterium tumefaciens*, interconvert between a stable Pfr state and a metastable Pr state [7].

Despite numerous studies on the structure and function of phytochromes, fundamental questions regarding the light-activation mechanism and its coupling to phytochrome function remain. For example, it is not fully understood how the structural changes in Pr and Pfr are triggered by the light-induced isomerization of the chromophore, although structural differences between Pr and Pfr have been identified in bacteriophytochrome [6]. Recent results on ultrafast proton-coupled isomerization in the phototransformation of phytochrome demonstrate how proton-coupled dynamics in the excited state of Pfr lead to a restructured hydrogen-bond environment in early Lumi-F, which is thought to be a trigger for downstream protein structural changes [4,5,8]. Moreover, several studies on the phytochrome have revealed that proton translocation plays a crucial role in the coupling of chromophore and protein conformational changes [9,10].

In addition to structural and ultrafast spectroscopic studies, time-resolved fluorescence anisotropy measurements in the picosecond to nanoseconds time range enable a variety of experiments in which fluorescent dyes can be used to monitor the conformational dynamics of proteins at a specific site [11,12,13,14,15]. In this respect, the fluorescence labeling of phytochromes [13,16] provides an opportunity to study local conformational changes in aqueous solution that would be otherwise hidden in crystal structure studies.

**Figure 1:**
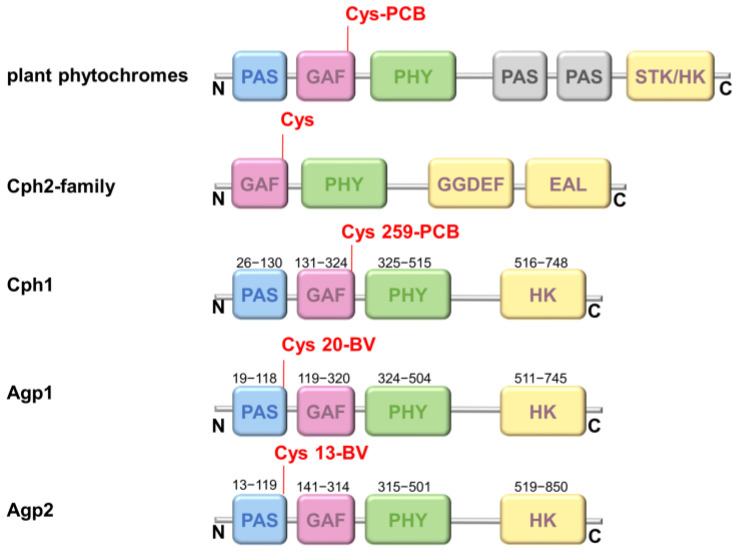
Protein domains in different phytochromes. The different photosensory domains PAS (blue), GAF (magenta), PHY (green), and the output domain (histidine kinase (HK) or similar (STK-HK: HK related domain; GGDEF-EAL: c-di-GMP turnover domain) (yellow) are shown according to the N- to C-terminal phytochrome sequence. Almost all phytochromes hold only a single chromophore-binding site at a conserved cysteine residue either at the PAS domain in the case of Agp1 and Agp2 or GAF domain in the case of plant phytochromes, Cph1, and Cph2.

**Figure 2:**
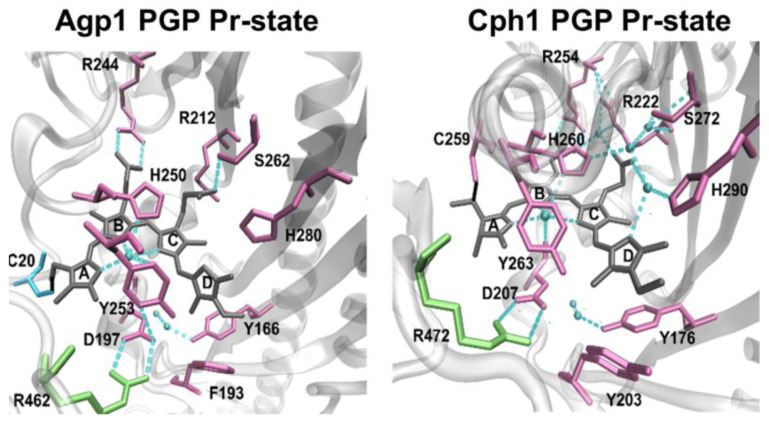
Chromophore-binding pocket of BV- and PCB-binding phytochromes: Agp1 PGP in Pr state, PDB 5I5L [18] and Cph1 in Pr state, PDB 2VEA [19].

Using this fluorescence-based approach, we test here the hypothesis that chromophore deprotonation is coupled to conformational changes in the different protein domains, i.e., whether long-range conformational changes exists and whether a long-range H-bonding network between the chromophore-binding pocket and the PHY domain controls the chromophore p*K*_a_ in Agp1, as shown for Cph1 [13]. The canonical (prototypical) phytochrome Agp1 differs from Cph1 in the bound bilin chromophore. While Cph1 and plant phytochromes bind phycocyanobilin (PCB), Agp1 binds biliverdin (BV). The domain structure of plant phytochromes, Cph1, Agp1 and some other phytochromes are shown in Figure 1 for comparison. The structure of the chromophore-binding pocket of the two phytochromes, Cph1 and Agp1 with the PCB and BV chromophore, respectively, is shown in Figure 2. Among others, the conserved tyrosines Y166 and Y253 in Agp1 and Y176 and Y263 in Cph1, as well as the conserved salt bridge between D197 (GAF) and R462 (PHY) in Agp1 and D207 and R472 in Cph1, whose cleavage is thought to be essential to interconvert from Pr to Pfr, are indicated. Via this strictly conserved aspartic acid in the chromophore-binding pocket, the chromophore connects to the PHY-tongue.

By using chromophore titrations and picosecond time-resolved fluorescence anisotropy measurements to observe structural dynamics in Agp1 at the site of fluorescence-sensor attachment, we answer the following questions: Does the modification in the PHY domain of Agp1 affect chromophore deprotonation, similar to what was observed in Cph1? Is the long-distance protonation-conformation coupling, as observed for Cph1 between the chromophore protonation state and the distant PHY domain, also present in Agp1 and correlated to protonation heterogeneity in the chromophore-binding pocket [13,17]?

We show that local conformational dynamics in the different photosensory domains and its coupling to the chromophore behaves differently in PCB-binding Cph1 and BV-binding Agp1 and is, thus, connected to a different long-range H-bonding network. This indicates phytochrome species-specific correlations between transient chromophore deprotonation and the photo-induced large global protein rearrangements and intramolecular signal transduction.

## 2. Results

### 2.1. Protein Variants, Their Spectroscopic Characterization and Fluorescein Labeling of the Photosensory Module

**Figure 3:**
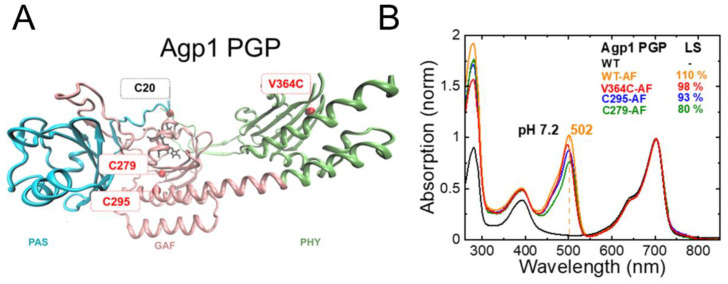
Agp1 PGP structure, single accessible cysteine mutants, and fluorescein-labeling. (**A**) Structural model of Agp1 PGP (PDB 5I5L) with accessible single cysteines for fluorescence labeling in the indicated in red (C279 and C295 as cysteines in the native sequence, and V364C as a cysteine introduced in the PHY domain), the PCB chromophore attached to Cys20 in black. To introduce single accessible cysteine, the following mutants were constructed: C297S/C295, C295S/C279, and C279S/C295S/V364C. The PAS, GAF, and PHY domains are colored in blue, pink, and green, respectively. The figure was generated with VMD. (**B**) Pr absorption spectrum of Agp1 PGP and IAF-labeled variants at pH 7.2, normalized to the chromophore peak at 702 nm; WT in black, WT-AF in orange, V364C-AF in red, C295-AF in blue, and C297-AF in green. Labeling was performed as described in Section 4 and the labeling stoichiometry (LS) was calculated according to Equation (1), which yielded LS of 110%, 98%, 93%, and 80% for WT-AF, V364C-AF, C295-AF, and C297-AF, respectively. In WT, the two cysteines C295 and C279 are partly accessible as evidenced by the LS below 200%. The maximum absorption wavelength of the fluorescein peak in WT is indicated. Conditions: 300 mM NaCl, 50 mM Tris, pH 7.2 at 20 °C.

Agp1-PGP (wild type, WT), Agp1-PGP-C279S/C295S, Agp1-PGP-C279S, Agp1-PGP-C295S, and Agp1-PGP-C279S/C295S/V364C (Figure 3A), were labeled with the pH-indicator dye 5-iodoacetamidofluorescein (5-IAF) to about 80–110%, yielding WT-AF, C279-AF, C295-AF, and V364C-AF. The corresponding UV–Vis absorbance spectra of the IAF-labeled and unlabeled Agp1-PGP samples are shown in Figure 3B and were used for calculating the labeling stoichiometry (LS) according to Materials and Methods. To test whether the introduced single labeling site (C279, C205, or V364C) is the only accessible labeling site for the fluorophore IAF in the respective Agp1 variant, we also performed IAF-labeling for the variant Agp1-PGP-C279S/C295S, which only contains the cysteine in position 20 for covalent binding of the BV chromophore.

**Figure 4:**
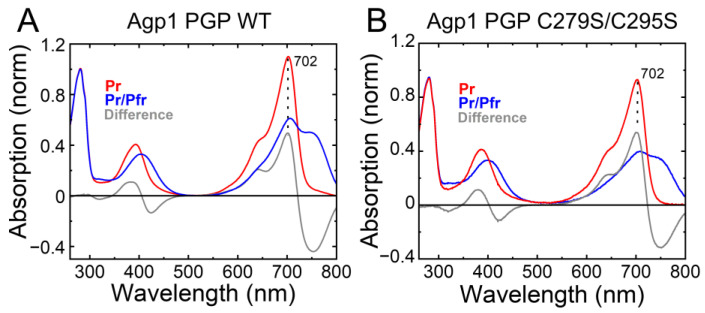
Characterization of the accessible cysteine-less base variant Agp1 PGP C279S/C295S. The UV–Vis absorption spectra in the Pr state (in red), after illumination in the Pr/Pfr state (in blue), and the difference spectra (in grey) are shown. The maximum absorbance wavelength of the Pr state peak is given. (**A**) Agp1 PGP WT. (**B**) Agp1 PGP C279S/C295S. Conditions: 300 mM NaCl, 50 mM Tris, pH 7.8 at 20 °C.

Indeed, IAF-labeling of Agp1-PGP-C279S/C295S resulted in only residual labeling of the protein, probably due to minor amounts of apoprotein in the sample. We further tested whether this base mutant C279S/C295S changes the photochromicity of Agp1. The UV−Vis absorption spectrum of Agp1-PGP C279S/C295S in Pr is identical to that of WT showing the same maximum absorption wavelength (λ_max_) of 702 nm. The similarity also holds true for the formation of the Pfr state after illumination, as shown in Figure 4. This indicates that replacing the two cysteines in the GAF domain by serine does not affect the chromophore-binding pocket.

### 2.2. pH-Dependence of UV–Vis Absorption of Agp1-PGP and Its Variants

In recent studies [13,17], we determined the PCB chromophore p*K*_a_ in Cph1-PGP and fluorescein-labeled Cph1 variants. As observed for Cph1 [13], fluorescein-labeling at sites distal to the bilin chromophore affected chromophore p*K*_a_ substantially. Such a long-range effect suggests the existence of a long-range H-bond network from the PHY domain to the chromophore-binding pocket [13]. Here, we investigate the pH-dependence of the Agp1-PGP absorption spectrum with and without labeling of the protein with fluorescein to test whether fluorescein-labeling of the different Agp1 domains affects the chromophore p*K*_a_.

**Figure 5:**
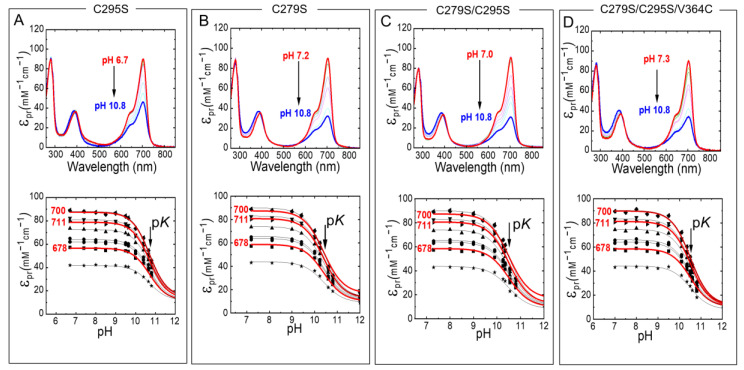
pH-dependent Pr absorption spectra of Agp1-PGP and its variants (top panel). (**A**) C295S, (**B**) C279S, (**C**) C279S/C295S, (**D**) C279S/C295S/V364C. The direction of the pH titration is indicated by the black arrow. The bottom panel shows the respective titration data at nine wavelengths 678 (■), 683.5 (●), 689 (▲), 694.5 (▼), 700 (⧫), 705.5 (◄), 711 (►), 716.5 (⬣), and 722 nm (★). Selected fit curves at 678, 700, and 711 nm are marked in red. The pH-dependence was fitted by the Henderson−Hasselbalch equation (Equation (2)) using Origin Pro 2019 (Origin Lab). The fit results are summarized in Table 1. The absorption of the deprotonated chromophore at high pH was estimated to 20% of the maximum absorption of the respective wavelength. Conditions: 300 mM NaCl, 50 mM Tris/HCl buffer for pH 6.2–9.5, or 100 mM Na_2_CO_3_/NaHCO_3_ buffer for pH 9.5–10.8, at 20 °C.

Absorption spectra of Agp1-PGP and its variants were measured at different pH (Figure 5). Titration curves from the absorbance changes at nine distinct wavelengths were used to calculate the p*K*_a_ values in the Pr state. In agreement with earlier studies of Agp1, the pH titration of Agp1-PGP WT yields only one p*K*_a_ of 10.70 ± 0.02. This p*K*_a_-value is slightly lower than the value of 11.1 determined earlier for Agp1 [20], probably due to slight variations in the protein purification or BV assembling protocols. In contrast to our findings in the canonical phytochrome Cph1 [13], we found only minor changes in the chromophore p*K*_a_ between Agp1-PGP WT and its variants (Table 1). The maximum change in chromophore p*K*_a_ was observed between Agp1-PGP WT and Agp1-PGP C279S/C295S/V364C with p*K*_a_ = −0.2. The corresponding labeling site in Cph1-PGP (C371) yielded a down-shift in chromophore p*K*_a_ of p*K*_a_ = −0.8 [13].

**Figure 6:**
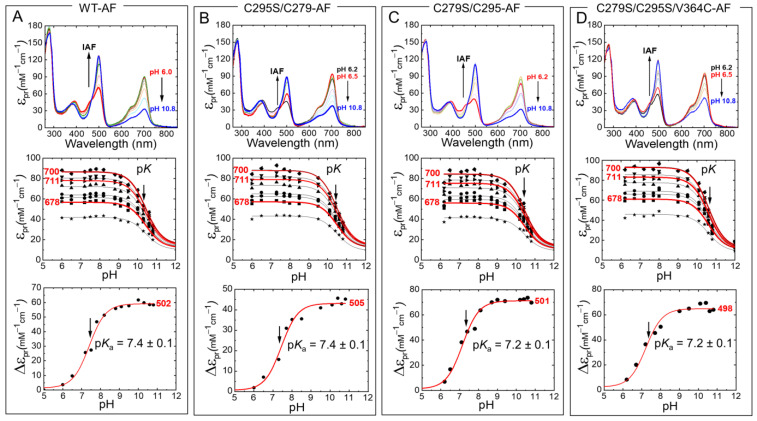
pH-dependent Pr absorption spectra of IAF-labeled Agp1-PGP and its variants (top panel). (**A**) WT-AF, (**B**) C295S/C279-AF, (**C**) C279S/C295-AF, (**D**) C279S/C295S/V364C-AF. The direction of the pH titration is indicated by the black arrow. The middle panel shows the respective titration data at nine wavelengths 678 (■), 683.5 (●), 689 (▲), 694.5 (▼), 700 (⧫), 705.5 (◄), 711 (►), 716.5 (⬣), and 722 nm (★). Selected fit curves at 678, 700, and 711 nm are marked in red. The bottom panel shows the respective titration data at the absorption maxima of the pH-sensitive absorption band of fluorescein with 502 nm (**A**), 505 nm (**B**), 501 nm (**C**), and 498 nm (**D**). The pH-dependence was fitted by the Henderson−Hasselbalch equation (Equation (2)) using Origin Pro 2019 (Origin Lab). The fit results for the middle panels are summarized in Table 1, the p*K*_a_ values for bound fluorescein are indicated in the figures. The absorption of the deprotonated chromophore at high pH was estimated to 20% of the maximum absorption of the respective wavelength. Conditions: 300 mM NaCl, 50 mM Tris/HCl buffer for pH 6.2–9.5, or 100 mM Na_2_CO_3_/NaHCO_3_ buffer for pH 9.5–10.8, at 20 °C.

Next, we measured the absorption spectra of fluorescein-labeled Agp1-PGP and Agp1-PGP variants at different pH values (Figure 6). Again, only a slight drop in the p*K*_a_ values was determined compared to the unlabeled WT (Table 1). For V364C-AF with the labeling position in the chromophore distant PHY domain, we calculated a change in the chromophore p*K*_a_ of only −0.1 pH units. In addition, we analyzed the pH-dependent absorbance band of bound fluorescein and determined the respective p*K*_a_ values of the fluorescein at the different sites (Figure 6, bottom panel). The p*K*_a_ values of the bound fluorescein are upshifted compared to the unbound fluorophore (p*K*_a_ = 6.5 [21]), indicating a similar negative surface potential at the labeling sites. The λ_max_ values of the fluorescein peak in the different variants, however, indicate different polarities at the labeling sites. The polarity was determined according to Alexiev et al. [22,23]. The fluorescein label in position 364 (Figure 3) located in the β-sheets in the chromophore distant PHY domain experiences the most hydrophilic environment with a λ_max_ = 498 nm, while position 297 in the GAF domain close to the chromophore-binding pocket (Figure 3) shows the most hydrophobic environment (λ_max_ = 505 nm).

Taken together, we observe a robust WT-like chromophore p*K*_a_ in the Agp1 variants investigated here, and upon modification of these variants with fluorescein in the GAF and PHY domain. This result is in contrast to our observation in Cph1 where a similar modification with fluorescein in the PHY domain at position C371 alters the chromophore p*K*_a_ by about −0.8 pH units, indicating a (direct or indirect) long-range H-bond network from the chromophore to the PHY domain in Cph1 [13]. Thus, we conclude from our results here that a similar long-range interaction from the PHY domain β-sheets to the chromophore is absent in Agp1. However, it is important to note that the chromophore p*K*_a_ of Agp1 is affected when the H-bond network within the chromophore-binding pocket is disrupted as shown previously for the mutant D1976A, which is part of the conserved Asp-Arg salt bridge (D197-R462 in Agp1, D207-R472 in Cph1) that connects the chromophore-binding pocket with the tongue (Figure 2). A drastic downshift in chromophore p*K*_a_ to about 7.6 was observed for this mutant [20]. This salt bridge and the hydrogen-bonding interaction of the aspartate were shown in simulations to affect the stabilization of the early/late Lumi-R intermediates featuring a more disordered chromophore-binding pocket [8].

### 2.3. pH-Dependence of Conformational Dynamics and Structural Constraints in Cph1 Constructs Using Time-Resolved Fluorescence Anisotropy

In prokaryotic phytochromes, transient bilin chromophore deprotonation after photoactivation is associated with conformational changes that culminate in de-/activation of the histidine kinase transmitter [24]. In Cph1, we found a direct correlation between chromophore deprotonation and pH-dependent conformational dynamics and flexibility of Cph1-PGP in its different domains in equilibrium experiments of the Pr-state [13].

We hypothesize that the absent long-range interaction between chromophore p*K*_a_ and PHY domain in Agp1 compared to Cph1 [13], as observed by the fluorescein-labeling experiments, also leads to an absent correlation between chromophore p*K*_a_ and pH dependence of conformational dynamics and protein flexibility in the Pr state.

Thus, we measured the time-resolved fluorescence depolarization of Agp1-bound fluorescein to detect the pico-nanoseconds structural dynamics of the protein. The anisotropy decay curve of the bound fluorophore provides information on global and local protein dynamics as well as on the protein structure and conformational changes [11,12,25].

**Figure 7:**
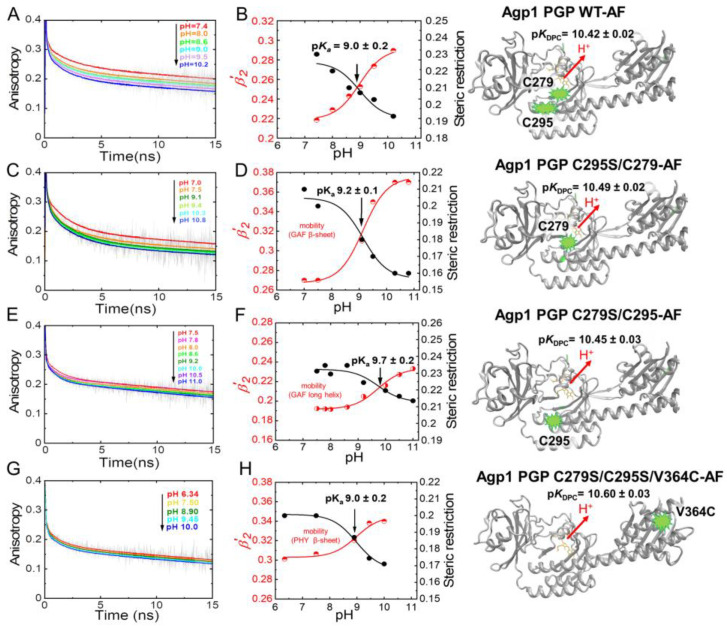
pH-dependent time-resolved fluorescence anisotropy data for Agp1 variants. (**A**,**C**,**E**,**G**) show the anisotropy decay curves (grey) and the respective fits (colored) at the different pH. (**B**,**D**,**F**,**H**) show the titrations curves of β-sheet flexibility expressed as the relative mobility *β*′_2_ = *β*_2_/(*β*_2_ + *β*_3_) (red data points), and of the steric restriction of the respective protein segment by the surrounding protein constituents expressed by *β*_3_ (black data points). The p*K*_a_ values of the pH-dependent conformational changes were obtained by global fitting of steric restriction and *β*′_2_ using Equation (2). The fit curves are shown. The respective p*K*_a_ values are given. (**A**,**B**) Agp1 PGP WT-AF, (**C**,**D**) Agp1 PGP C295S/C279-AF, (**E**,**F**) Agp1 PGP C279S/C295-AF, and (**G**,**H**) Agp1 PGP C279S/C295S/V364C-AF. The chromophore p*K*_a_ values are depicted alongside with the structural models at the right. The anisotropy fit values are summarized in Table 2 and Table 3. Conditions: 300 mM NaCl, 50 mM Tris, at 19 °C.

Figure 7 shows the anisotropy curves at five to eight different pH values between pH 6.3 and pH 11 for Agp1-WT-AF and the fluorescein-labeled variants. In WT-AF, the fluorescein reporter groups are located at the two native cysteines, one in the β-sheet region in the GAF domain (C279) and the other in the long helix part (C295) close to the chromophore-binding pocket (Figure 3). Data analysis revealed relatively small changes in the rotational correlation times for the β-sheets with 0.9 ± 0.09 ns as a function of pH (Table 2), whereas the amplitudes of the anisotropy decay components and, in particular, the steric restriction, change significantly with pH (Table 2). Titration curves from the anisotropy amplitude changes were used to calculate the p*K*_a_-value (Figure 7B), which was found to be 9.0 ± 0.2 in WT-AF. Next, we directly observed the pH-dependent GAF β-sheet mobility in C295S/C279-AF, i.e., the conformational space/steric restriction of β-strand movement, and found a similar p*K*_a_ = 9.2 ± 0.1 (Figure 7D). In comparison to the GAF β-sheet mobility, the long helix (spine) in the GAF domain exhibits a different pH dependence of its mobility as revealed in C279S/C295-AF. A faster rotational correlation time of this segment (0.5 ns at pH 7.5) correlates with a reduced pH-dependent change in conformational space and an increase in the corresponding p*K*_a_ to 9.7 ± 0.2 (Figure 7F). When investigating the conformational dynamics in the PHY domain β-sheet using V364C-AF, a slower rotational correlation time for the β-sheets with 1.4 ns at pH 7.5 was found. The change in pH-dependent conformational space was smaller than for the GAF domain β-sheets, but a similar p*K*_a_-value of 9.0 ± 0.2 was found (Figure 7H, Table 2).

**Figure 8:**
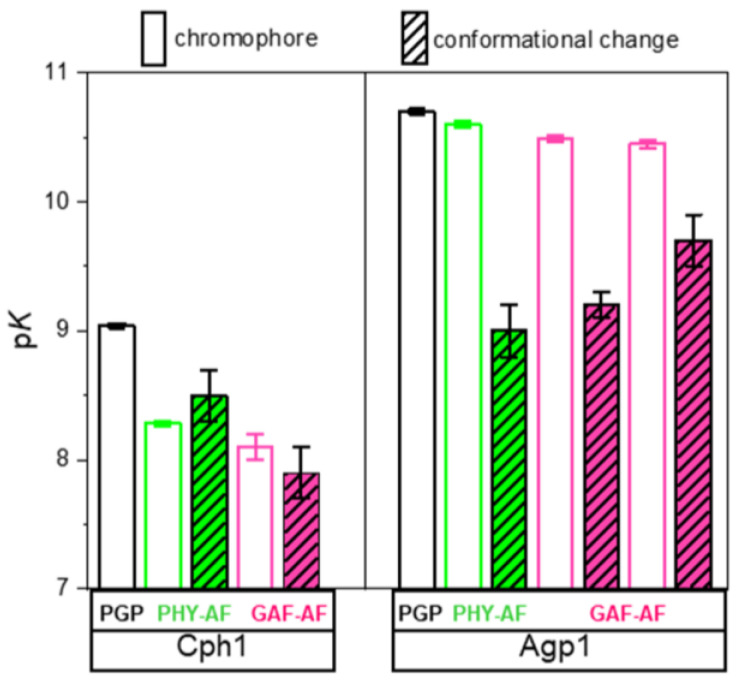
Comparison between chromophore p*K*_a_ and pH-dependent conformational change in the PHY and GAF domain for Cph1 and Agp1. Data from Figure 7, Table 3 and reference [13].

The respective p*K*_a_ values of chromophore deprotonation (p*K*_DPC_) are depicted alongside the structural models on the right side of Figure 7. The comparison between the p*K*_a_ values of chromophore deprotonation and the p*K*_a_ value of the different domain mobilities shows no correlation (Table 3, Figure 8), as hypothesized above.

## 3. Discussion

A number of fundamental questions regarding the mechanism of phytochrome light activation and subsequent signal transfer remain unanswered. Although structural differences between Pr and Pfr have been identified in bacteriophytochrome [6], it is not fully understood how these changes are triggered by the light-induced isomerization of the chromophore. Also, the mechanism of signal transmission from the chromophore-binding pocket to the transmitter region is still unclear. While a recent femtosecond X-ray laser study [4] focused on ultrafast collective changes in the chromophore-binding pocket, including protein backbone and water movements around the chromophore, a theoretical study [26] shed new light on the activation mechanism leading to the large structural change enabling signal transduction. The latter study indicates a large structural relaxation in solution compared to the crystal structure and an internal re-organization of the PHY domain upon light-activation. This internal re-organization, i.e., rotation of the PHY domain, is thought to be a consequence of the tongue re-folding from the β-sheet to the α-helical conformation in Pfr after chromophore isomerization, leading to a new conformation of the long helix through constraint of the H-bonds to the adjacent GAF and PHY domains, and thus to the large-scale conformational change enabling transmitter activation. Tongue re-folding was experimentally shown to occur with the last transition between the intermediate Meta-R and the Pfr state in *Deinococcus radiodurans* phytochrome (DrBphP) [27]. Together with the new understanding of the phytochrome activation pathways in the study by Macaluso et al. [26], this study underlines the importance of solution experiments that provide structural information.

An open question also concerns the de-/protonation of the chromophore during the photoconversion from Pr to Pfr. While theoreticians focus on the chromophore protonation in the Pr and Pfr ground or excited states [28,29], it is generally accepted that proton (s) are released and subsequently taken up by the protein upon the transition from Pr to Pfr, as shown in spectroscopic experiments [9,10]. However, the exact correlation with intermediate states, and whether tongue re-folding is causally associated with protonation dynamics remains elusive.

The pH titration of phytochromes indicates that chromophore absorption is highly pH-dependent in the Pr-state as found for many bacteriophytochrome species in the range between pH 6 and 10 [10,20], also recently shown in detail for Cph1 [17]. This pH-dependent difference in absorption can be explained by chromophore deprotonation, since deprotonation of the bilin chromophore nitrogen results in lowering of the chromophore extinction coefficient [30,31]. Employing such equilibrium titrations in fluorescently labeled Cph1 in position 371 we obtained an unexpected result. We found that both labeling with fluorescein and the mutation C371S, located in the chromophore-distant PHY domain, lead to a substantial downshift of the chromophore p*K*_a_. Our findings were rationalized in our previous publication as follows [13]. We assumed a conformational change, induced by the mutation/labeling with fluorescein, that propagates through the arrangement of hydrogen bonds [32] from the PHY domain β-sheet to the chromophore-binding pocket. This hypothesis was supported by the correlation of the pH dependence of the local conformational change in the distant PHY domain with the chromophore p*K*_a_ [13].

Here, we investigated whether a similar long-range rearrangement of hydrogen bonds exist and would lead to a correlation of chromophore p*K*_a_ and pH-dependent conformational flexibility in the distant PHY domain of another canonical phytochrome, namely Agp1 which, however, binds the biliverdin chromophore in the PAS domain, most distant to the PHY domain (Figure 1). We constructed the mutant Agp1-PGP V346C to test this hypothesis. To our surprise, neither a mutation-dependent change in chromophore p*K*_a_ nor a correlation between chromophore p*K*_a_ and pH-dependent domain mobility, was observed for Agp1 (Figure 8). This result is also in line with the absence of intersubunit distance changes as found for Agp1 using PELDOR measurements [33].

We conclude the following: (i) Agp1, which binds BV to position C20 (PAS) instead of PCB to position C259 (GAF) in Cph1, behaves differently compared to Cph1 when the analogous position to C371 in the PHY domain is mutated (V346C) or labeled with fluorescein. No long-distance effects on the chromophore deprotonation were observed in Agp1. (ii) The absence of these long-distance effects correlates with an absence of the coupling between pH-dependent conformational changes in the distant PHY domain and the chromophore p*K*_a_. (iii) While the properties within the chromophore-binding pocket and the activation mechanisms seem to be highly conserved [8], activation pathways from the chromophore-binding pocket to the PHY domain may vary and were suggested to allow phytochrome to exploit redundant routes for its activation [26].

The latter is supported by experimental results where substitution of the conserved tyrosine Y263 to phenylalanine resulted in a decoupling between protein conformation (Pfr) and chromophore state (Pr) in DrBphP [34]. Similarly, the Y263S mutation in the canonical Cph1 phytochrome resulted in a block of the Pr to Pfr photoconversion as evidenced by time-resolved absorption experiments [35].

Regarding the mechanism of signal transmission from the chromophore-binding pocket to the transmitter region, we now provide further experimental evidence that various mechanisms may exist that couple chromophore deprotonation to the mobility of the PHY domain which could translate into the global conformational change. We speculate that the long-range coupling observed in the canonical phytochrome Cph1 harboring the PCB chromophore and the absence of this coupling in the canonical Agp1 with the BV chromophore might be connected to the two different chromophores, and thus probably also to the protonation heterogeneity observed in the Pr state of the PCB-binding Cph1 [13,17] but not in the BV-binding Agp1.

## 4. Materials and Methods

### 4.1. Phytochrome Mutagenese, Expression and Purification

The photosensory module of wild-type Agp1 (residues 1–504 with a C-terminal His6-tag, Agp1-PGP), and the constructs Agp1-PGP-C279S/C295S, Agp1-PGP-C279S, Agp1-PGP-C295S, and Agp1-PGP-C279S/C295S/V364C were expressed in E. coli, the cells lysed, and the soluble fraction purified following established methods [19]. The apoprotein was expressed and assembled with BV according to established protocols [36]. The plasmid agro1-M15-9N, based on the original plasmid [18] was kindly provided by Prof. Hildebrand (TU Berlin). This plasmid and the plasmid without deletion of the first 9 N-terminal amino acids were used for the mutagenesis. Site-directed mutagenesis of Agp1-PGP was carried out according to the Quik Change^TM^ mutagenesis protocol (Agilent Technologies, Santa Clara, CA, USA). The required enzymes were purchased from New England Biolabs, Ipswich, MA, USA.

### 4.2. Phytochrome Labeling with IAF

The photosensory module Agp1-PGP, Agp1-PGP-C279S/C295S, Agp1-PGP-C279S, Agp1-PGP-C295S, and Agp1-PGP-C279S/C295S/V364C were labeled with a 10-fold molar excess of 5-iodacetamidfluorescein (IAF, Invitrogen Molecular Probes, Waltham, MA, USA) and 2-fold molar excess of DTT in 50 mM Tris pH 7.8, 150 mM NaCl for 8 h at room temperature in the dark. Gel filtration (Sephadex G-25 fine, GE Healthcare, Chicago, IL, USA) was used to remove unlabeled dye (adapted from [16,25]). The molar labeling stoichiometry was determined by

(1) $$\frac{c_{Label}}{c_{Protein}}=\left(\frac{\Delta A_{Label}}{\varepsilon_{Label}}\right)\left(\frac{\varepsilon_{Protein,Pr}}{A_{Protein,Pr}}\right)$$

The absorbance *A_Protein,Pr_* was measured at λ_max_ = 702 nm. The corresponding extinction coefficient is *ε_Protein,Pr_* = 90,000 M^−1^ cm^−1^ [37]. The measurements were performed under green light to avoid photoconversion of Pr. The absorbance difference between the labeled and unlabeled samples, Δ*A_L_*, at λ_max_ (500 nm) is the absorbance of the fluorescein label. The corresponding extinction coefficient of IAF at λ_max_ is *ε_Label_* = 67,000 M^−1^ cm^−1^ at pH 7.0 and *ε_Label_* = 77,000 M^−1^ cm^−1^ at pH 7.8 (Invitrogen Molecular Probes). Covalent binding of IAF to Agp1-PGP and its variants, and removal of excess unlabeled dye, was verified by Tricine-SDS-PAGE using the fluorescence band of IAF-labeled Agp1-PGP.

### 4.3. UV–Vis Spectroscopy, pH-Titration and pK_a_ Determination

UV–Vis absorbance spectra were measured with a Shimadzu UV2450 spectrometer (Shimadzu, Kyoto, Japan). pH titration was carried out at 20 °C in 50 mM Tris-citrate buffer including 15 mM NaCl. The pH was adjusted with small aliquots of NaOH and HCl in the pH range from 6.5 to 10.3. The Pr state was generated by saturating irradiation using a 735 nm LED (Conrad Electronics, Hirschau, Germany). The experiments were performed with aliquots in parallel, with different aliquots used in different pH ranges. The pH was measured before each experiment with a microelectrode (Metrohm, Herisau, Switzerland) in a measurement volume of 60 µL. The following controls were carried out: The samples were tested at the extreme pH values to identify the pH range in which the protein was stable for the time of the measurement. For pH values below pH 6 and above pH 11 immediate aggregation was identified by UV–Vis absorption. Also, before each TCSPC experiment, the absorption spectrum was checked. Only low intensity background light was used during the measurements to avoid photoconversion of Pr. The absorbance values were scaled to the extinction coefficient ε_700nm_ of Pr at pH 7.8 (90,000 M^−1^ cm^−1^) for presentation. The pH-titration curves were generated from the respective absorbance values at nine wavelengths from 678–722 nm. A global fit with the Henderson−Hasselbalch equation (Equation (2)) was performed

(2) $$\Delta A\left(pH\right)=\;\Delta A_{max}/\left(1+10^{\left(pK-pH\right)}\right)$$

### 4.4. Time-Resolved Fluorescence Spectroscopy

The fluorescence anisotropy decay measurements were performed as described in [14,25,38]. Briefly, the sample was measured using a picosecond Ti:sapphire laser system (Millenia vs and Tsunami, Spectra Physics, Milpitas, CA, USA) and a microchannel plate detector (model #R3809U, Hamamatsu, Shizuoka, Japan) in a time-correlated single-photon counting (TCSPC) setup with TCSPC card SPC-830 (Becker & Hickl, Berlin, Germany). Fluorescence excitation was at 488 nm and emission was detected using a 515 nm long-pass filter (GG515). The fluorescence lifetime traces were recorded in 1024 time channels with an instrument response function (IRF) of 30–40 ps. The instrument response function (IRF) of the TCSPC setup was determined at the corresponding wavelengths with a colloidal silica solution as the scattering material (LUDOX, Grace). Fluorescence decay traces were fitted using a sum of exponentials with an amplitude *α_i_* and a decay constant *τ_i_* after deconvolution with the IRF

(3) $$I\left(t\right)=\overset{n}{\underset{i=1}{\sum }}\alpha_{i}e^{-t/\tau_{i}}$$

Fluorescence anisotropy time traces *r*(*t*) were calculated by measuring parallel $I_{∥}\;\left(t\right)$ and perpendicular $I_{⊥}\;\left(t\right)$ polarized fluorescence intensity compared to the excitation polarization using

(4) $$r\left(t\right)=\frac{I_{∥}\left(t\right)-I_{⊥}\left(t\right)}{I_{∥}\left(t\right)+2I_{⊥}\left(t\right)}$$

Anisotropy fit data were acquired using the following model function in the software Globals (Laboratory for Fluorescence Dynamics, University of California, Irvine, CA, USA)

(5) r(t)=∑ i=13βie−tϕi

with the initial anisotropy (at *t* = 0: *r*_0_ = (*β*_1_ *+ β*_2_ *+ β*_3_). The rotational correlation times *ϕ*_1_ and *ϕ*_2_ describe the rotational motion of the label and the protein segment to which the labeled is attached. *ϕ*_3_ describes the rotational diffusion of the whole system. The amplitudes *β*_1_ and *β*_2_ indicate the degree of depolarization of the anisotropy decay components with the correlation times *ϕ*_1_ and *ϕ*_2_, respectively. If the rotational correlation time of the last decay component is much slower than the lifetime of the fluorescent probe, the anisotropy decays virtually to a constant end value r_∞_ in the ns time range of the measurements that is determined by the lifetime of the fluorescent probe. The value of *r*_∞_, or the amplitude of the slowest anisotropy decay component, in our case *β*_3_, represents a measure of the degree of steric hindrance by the protein surface.

The conformational space of the protein segment is expressed as relative mobility using

(6) $$\beta_{2}^{\prime }=\beta_{2}/\left(\beta_{2}+\beta_{3}\right)$$

## Figures and Tables

**Table 1:** Comparison of p*K*_a_ values of biliverdin chromophore deprotonation (p*K*_DPC_) of the different AF-labeled and unlabeled Agp1-PGP variants from Figure 5 and Figure 6. The standard error is given (± SD).

Sample	Chromophore p*K*_DPC_	p*K*_DPC_ ^a^
Agp1 PGP WT	10.70 ± 0.02	-
Agp1 PGP C295S	10.70 ± 0.01	0 ± 0.03
Agp1 PGP C279S	10.40 ± 0.02	−0.3 ± 0.03
Agp1 PGP C279S/C295S	10.51 ± 0.02	−0.19 ± 0.04
Agp1 PGP C279S/C295S/V364C	10.50 ± 0.02	−0.20 ± 0.04
Agp1 PGP WT-AF	10.42 ± 0.02	−0.28 ± 0.04
Agp1 PGP C295S/C279-AF	10.49 ± 0.02	−0.21 ± 0.04
Agp1 PGP C279S/C295-AF	10.45 ± 0.03	−0.25 ± 0.05
Agp1 PGP C279S/C295S/V364C-AF	10.60 ± 0.03	−0.09 ± 0.05

^a^ *K*_DPC_ values were calculated for the different constructs with respect to PGP WT. The standard error is given.

**Table 2:** Time-resolved anisotropy fit results of Agp1-PGP-AF variants. WT-AF (A), C279-AF (B), C295-AF (C), V364C-AF (D). r_0_ is the initial anisotropy and the amplitudes *β*_1_ and *β*_2_ indicate the degree of depolarization of the anisotropy decay components with the correlation times *ϕ*_1_ and *ϕ*_2_, respectively. The tumbling of the whole protein is characterized by *ϕ*_3,_ the corresponding steric restriction by *β_3_*. The reduced χ^2^ (χ_red_^2^) is given as a measure of the goodness of the fit.

**(A) Agp1 PGP WT-AF**								
pH	r_0_	*ϕ*_1_ (ns)	*ϕ*_2_ (ns)	*ϕ*_3_ (ns) ^a^	*β*_1_	*β*_2_	*β*_3_	χ_red_^2^
7.4	0.35	0.10	1.05	30.0	0.052	0.065	0.232	0.95
8.0	0.35	0.08	0.82	30.0	0.063	0.066	0.220	0.93
8.6	0.34	0.08	0.82	30.0	0.058	0.071	0.211	0.93
9.0	0.34	0.11	0.91	30.0	0.066	0.066	0.207	0.92
9.5	0.34	0.07	0.87	30.0	0.062	0.076	0.202	1.00
10.2	0.33	0.13	0.92	30.0	0.059	0.079	0.192	0.93
**(B) Agp1 PGP C279-AF**								
pH	r_0_	*ϕ*_1_ (ns)	*ϕ*_2_ (ns)	*ϕ*_3_ (ns) ^a^	*β*_1_	*β*_2_	*β*_3_	χ_red_^2^
7	0.34	0.10	1.10	30	0.048	0.078	0.214	0.96
7.5	0.34	0.31	2.25	30	0.070	0.071	0.199	1.04
9.1	0.34	0.23	1.17	30	0.078	0.082	0.180	0.87
9.5	0.34	0.17	1.10	30	0.065	0.098	0.174	1.06
10.3	0.34	0.18	1.17	30	0.077	0.097	0.161	1.02
10.8	0.34	0.27	1.5	30	0.088	0.093	0.160	0.96
**(C) Agp1 PGP C295-AF**								
pH	r_0_	*ϕ*_1_ (ns)	*ϕ*_2_ (ns)	*ϕ*_3_ (ns) ^a^	*β*_1_	*β*_2_	*β*_3_	χ_red_^2^
7.5	0.34	0.04	0.50	30	0.054	0.055	0.231	1.01
7.8	0.34	0.04	0.41	30	0.049	0.056	0.235	0.96
8.0	0.34	0.04	0.50	30	0.056	0.054	0.229	0.95
8.6	0.34	0.05	0.51	30	0.049	0.056	0.235	0.95
9.2	0.34	0.04	0.52	30	0.058	0.058	0.224	1.01
10.0	0.34	0.06	0.60	30	0.060	0.060	0.2196	0.96
10.5	0.34	0.07	0.70	30	0.060	0.063	0.2162	0.98
11.0	0.34	0.05	0.60	30	0.061	0.066	0.2135	0.94
**(D) Agp1 PGP V364C-AF**								
pH	r_0_	*ϕ*_1_ (ns)	*ϕ*_2_ (ns)	*ϕ*_3_ (ns) ^a^	*β*_1_	*β*_2_	*β*_3_	χ_red_^2^
6.3	0.34	0.15	1.40	30	0.049	0.086	0.204	0.96
7.5	0.34	0.15	1.40	30	0.059	0.085	0.199	0.94
8.9	0.34	0.15	1.35	30	0.065	0.088	0.187	1.01
9.5	0.34	0.15	0.60	30	0.076	0.089	0.174	0.97
10.0	0.34	0.15	1.70	30	0.080	0.088	0.171	0.98

^a^ *ϕ*_3_ (ns) is kept constant at 30 ns for fitting.

**Table 3:** Comparison of p*K*_a_ values of biliverdin chromophore deprotonation (p*K*_DPC_) of the different AF-labeled Agp1-PGP variants and the p*K*_a_ values of protein conformational changes in the different domains. The standard error is given (± SD).

Sample	Chromophore p*K*_DPC_	p*K*_a_ of Conformational Change
Agp1 PGP WT-AF (labeling in GAF)	10.42 ± 0.02	9.0 ± 0.2
Agp1 PGP C295S/C279-AF (labeling in β-sheet of GAF)	10.49 ± 0.02	9.2 ± 0.1
Agp1 PGP C279S/C295-AF (labeling in long helix of GAF)	10.45 ± 0.03	9.7 ± 0.2
Agp1 PGP C279S/C295S/V364C-AF (labeling in β-sheet of PHY)	10.60 ± 0.03	9.0 ± 0.2

## Data Availability

The data presented in this study are openly available in FigShare at 10.6084/m9.figshare.21647348.

## Sample Availability

Samples of the compounds are available from the authors.
